# Advances in the Immunology and Genetics of Leprosy

**DOI:** 10.3389/fimmu.2020.00567

**Published:** 2020-04-16

**Authors:** Zihao Mi, Hong Liu, Furen Zhang

**Affiliations:** Shandong Provincial Hospital for Skin Diseases & Shandong Provincial Institute of Dermatology and Venereology, Shandong First Medical University & Shandong Academy of Medical Sciences, Jinan, China

**Keywords:** leprosy, innate immunity, adaptive immunity, genetic association, variant

## Abstract

Leprosy, a disease caused by the intracellular parasite *Mycobacterium leprae* or *Mycobacterium lepromatosis*, has affected humans for more than 4,000 years and is a stigmatized disease even now. Since clinical manifestations of leprosy patients present as an immune-related spectrum, leprosy is regarded as an ideal model for studying the interaction between host immune response and infection; in fact, the landscape of leprosy immune responses has been extensively investigated. Meanwhile, leprosy is to some extent a genetic disease because the genetic factors of hosts have long been considered major contributors to this disease. Many immune-related genes have been discovered to be associated with leprosy. However, immunological and genetic findings have rarely been studied and discussed together, and as a result, the effects of gene variants on leprosy immune responses and the molecular mechanisms of leprosy pathogenesis are largely unknown. In this context, we summarized advances in both the immunology and genetics of leprosy and discussed the perspective of the combination of immunological and genetic approaches in studying the molecular mechanism of leprosy pathogenesis. In our opinion, the integrating of immunological and genetic approaches in the future may be promising to elucidate the molecular mechanism of leprosy onset and how leprosy develops into different types of leprosy.

## Introduction

Leprosy is an ancient disease caused by *Mycobacterium leprae* or *Mycobacterium lepromatosis* infection, which mainly impairs skin and peripheral nerves and can even result in disability (1). The pathogens of leprosy have accompanied and affected humans for more than 4,000 years (2, 3), and over 200,000 new cases of leprosy are still reported each year worldwide (4) despite the application of multidrug therapy by the Word Health Organization. Because of the severe consequences caused by leprosy, including appearance changes and disabilities, leprosy is still a significant public health issue, especially in countries such as Brazil, India, and Indonesia, where the disease is still prevalent.

As the clinical manifestation of leprosy presents as a spectrum, it has long been considered an attractive model by immunologists to study the interaction between immune response and infection. Based on the different immune responses observed in patient lesions, leprosy can be categorized into five groups: tuberculoid (TT), borderline tuberculoid (BT), borderline borderline (BB), borderline lepromatous (BL), and lepromatous (LL) (5). The World Health Organization classifies leprosy clinically as multibacillary and paucibacillary, according to the number of skin lesions and nerve involvement (6). During the chronic infectious course, the immune-mediated acute inflammatory episodes called leprosy reactions frequently occurred. Leprosy reactions can be classified into two major types: type 1 reaction (T1R) or reversal reaction occurring mostly in unstable borderline patients (BT, BB, BL) and LL patients and type 2 reaction (T2R) or erythema nodosum leprosum (ENL) occurring mostly in BL and LL patients (7, 8). Therefore, leprosy is considered as an ideal disease model by immunologists to investigate the interrelation between pathogen load in infection and the differential immune responses of the host. Nevertheless, the pathogenesis of leprosy remains ambiguous due to the lack of an ideal animal model for this disease.

Leprosy is one of the most stigmatized diseases worldwide and was once thought to be a punishment from God on sinful persons due to the fact that only specific people developed this disease. In fact, for many infectious diseases, a common feature is that only a portion of the individuals who are exposed to the pathogens are actually infected and develop clinical symptoms, and genetic factors of the hosts have long been considered a major contributor to variances in susceptibility (9). In the case of leprosy, it has been estimated that just 5% of exposed individuals are successfully infected, of whom only 20% actually develop leprosy (10). Since the late 1900s, studies have shown that it is the genetic background and not God's punishment that makes infected individuals develop leprosy (11, 12). In the 21st century, studies using modern genetic approaches such as candidate gene association studies (CGASs) and genome-wide association studies (GWASs) have gradually confirmed that the host genetic background contributes greatly to the development of leprosy, and many leprosy-associated variants or genes have been reported. Most leprosy-associated genes are immune related, which is consistent with the finding that leprosy is caused by infection with pathogens.

Now it is clear that both the genetic background and the immune response of the host play essential roles in the development and manifestation of leprosy. Here, we firstly review studies on the responses of innate and adaptive immune cells in leprosy. We then summarize the leprosy-associated genes discovered by candidate gene and GWASs. Additionally, we proposed the combination of immunological and genetic studies to elucidate the underlying mechanisms of leprosy onset and development.

## Leprosy as an Infectious Disease

### Innate Immune Responses in Leprosy

#### Macrophages

As a key part of innate immunity and the major host of leprosy pathogens, macrophages have long been the focus of leprosy research. Macrophages in skin lesions of leprosy were dichotomously categorized into two types, the M1 type and M2 type. Epithelioid macrophages exhibiting M1 phenotype (CD68+CD163-) predominantly present in granulomas of TT patients, whereas macrophages in LL granulomas are foamy and mainly exhibit the M2 phenotype (CD68+CD163+) (13). Recently, de Sousa et al. (14) also characterized the presence of M4 macrophages in TT and LL lesions using double staining with markers of CD68 and MRP8. The expressions of both markers were stronger in LL than in TT lesions (14).

Although the differential polarization of macrophages in leprosy lesions is well-characterized, the intrinsic mechanisms of macrophage divergence in leprosy lesions are largely unknown. Interleukin (IL)-10, a key cytokine present in LL lesions, was shown to induce the phagocytosis program in human peripheral blood mononuclear cell (PBMC)-derived macrophages, whereas IL-15, which is abundant in TT lesions, triggered the vitamin D-dependent antimicrobial pathway (15). Using a co-culture system consisting of endothelial cells and monocytes, Kibbie et al. (16) found that unstimulated endothelial cells could trigger monocytes to become M2 macrophages, while endothelial cells stimulated by interferon (IFN)-γ or certain drugs induced the differentiation of monocytes to M1 macrophages in a Jagged1 (JAG1)-dependent manner (16). This study not only showed how macrophage divergence occurred at the site of infection but also provided new clues for intervening in intracellular infections. Moreover, a recent study focusing on miRNAome expression in leprosy physiopathology found that miR-34a-5p which controlled *JAG1* expression was upregulated in lepromatous leprosy (L-lep) lesions (17). Thus, downregulation of *JAG1* by miR-34a-5p allows the differentiation of M2 macrophage in L-lep, which is consistent with the discovery of a JAG1-dependent M1 macrophage differentiation (16). These studies demonstrate that cytokines, microRNA, and the microenvironment at the site of infection may play roles in macrophage divergence. Nonetheless, the reason these cytokines are differentially expressed in different types of leprosy remains mysterious, despite the exciting results of these two studies.

By comparing the expression of genes in polar forms of leprosy, three macrophage-related antimicrobial pathways have been found to function in the innate immune response to leprosy. As mentioned above, the vitamin D-dependent antimicrobial pathway has been characterized in TT leprosy (15). Differential expression of the miRNAome between TT and LL lesions found that microRNA hsa-mir-21 was mostly upregulated in LL lesions (18). Further analysis indicated that hsa-mir-21 could inhibit the expression of two vitamin D-dependent antimicrobial peptides by downregulating the Toll-like receptor 2/1 (TLR 2/1) pathway and upregulating IL-10 (18), which clearly demonstrated the interaction between leprosy pathogens and macrophage antimicrobial activity. In addition, other miRNAs targeting *TLR4* and *IL15R*, which also regulated the vitamin D-dependent antimicrobial pathway, were also observed to be upregulated in L-lep, indicating the inhibition of vitamin D-dependent antimicrobial pathway (17). These studies repeatedly demonstrated the role of vitamin D-dependent antimicrobial pathway in the control of *M. leprae* infection.

Silva et al. (19) systematically analyzed the expression of autophagy genes in tuberculoid leprosy (T-lep) and L-lep lesions. Autophagy genes were found to be significantly upregulated in T leprosy, whereas the autophagic flux was impaired in L-lep but could be restored by IFN-γ or rapamycin (19). This study suggested that autophagy was an innate response of macrophages to control *M. leprae*. Even more, autophagy was also suggested to play an important role in T1R in multibacillary leprosy. In a study of multibacillary leprosy (BL and LL), the researchers found that autophagy was downregulated in patients who developed T1R in the future compared to patients who did not develop T1R (20). And the authors also demonstrated a significantly higher level of IL-1β in the serum of T1R group months before T1R onset, suggesting IL-1β as a potential marker for T1R prediction (20).

Another canonical antimicrobial pathway, the nitric oxide (NO) antimicrobial pathway, was also studied by a quantitative analysis of inducible NO synthase (iNOS) expression in polar forms of leprosy (21). These results showed that the expression of iNOS in the LL form was significantly higher than that in the TT form, and a linear correlation was also observed between iNOS and CD68 (21). Additionally, an increased expression of iNOS was also observed in T1R patients compared to non-reactional patients, indicating the activation of macrophage in T1R (22, 23). It is possible that *M. leprae* could induce the expression of iNOS, but the NO antimicrobial pathway alone cannot control *M. leprae*. This view was confirmed by a report of milestone significance (24). Using a zebrafish model, the authors clearly showed that phenolic glycolipid I (PGL-I) of *M. leprae* could induce the expression of NOS and increase the production of reactive nitrogen species, which then injured axons by impairing mitochondria and inducing demyelination (24). Thus, the NO antimicrobial pathway cannot control *M. leprae* and is in fact potentially a key inducement of nerve damage in leprosy. Most recently, studies found that phenolic glycolipid I (PGL-I) shaped the innate immune response not only in macrophages but also in polymorphonuclear neutrophils (PMNs) and dendritic cells (DCs) (25). The interaction of PGL-I with CR3 promoted the invasion of the bacteria into these innate cells and selectively increased the production of IL-2 by DCs, IL-10 by PMNs, and IL-1β by macrophages, respectively, through the CR3–Syk–NFATc axis (25). These studies demonstrated how the virulence factor of *M. leprae* PGL-I shaped the innate immune responses of innate cells, which may eventually effect the clinical symptoms of leprosy.

In a genome-wide study of mRNA expression in leprosy, mRNA of *AKR1B10* was observed to be overexpressed in T2R lesions (26). Further immunohistochemistry investigation confirmed the overexpression of AKR1B10 in T2R and showed that *AKR1B10* was principally expressed by macrophage in leprosy lesions (27). Due to the unknown function of AKR1B10 in leprosy, the authors could not determine whether AKR1B10 overexpression was an event accompanying T2R or contributed to T2R development. Nonetheless, AKR1B10 expression was a potential biomarker of T2R.

#### Dendritic Cells

DCs are professional antigen-presenting cells and can process and present antigens to T cells. Considering the essential roles of DCs in cell-mediated immunity, DCs were thought to play roles in leprosy pathogenesis and different types of DCs have been examined in leprosy by several studies (Table 1).

**Table 1:** The expression of different DCs in leprosy.

**DC type (markers used)**	**DC location studied**	**Expression of markers**	**References**
LCs (CD1a)	Epidermis	Higher expression in T-lep lesions, lower expression in L-lep lesions	(28)
LCs (CD1a)	Epidermis	Higher expression in T-lep lesions, lower expression in L-lep lesions	(29)
LCs (CD1a)	Epidermis	No significant difference between indeterminate leprosy lesions and normal skin	(30)
LCs (CD1a, CD207)	Epidermis	Higher expression in T-lep lesions, lower expression in L-lep lesions	(31)
pDCs (CD123)	Dermal granulomas	Higher expression in type 1 reaction lesions, lower expression in L-lep lesions	(32)
pDCs (CD123)	Dermis	Insignificant expression of CD123 in indeterminate leprosy lesions and normal skin	(30)
pDCs (CD123)	Dermis	Higher expression in T-lep lesions, lower expression in L-lep lesions	(31)
DDs (FXIIIa)	Dermis	Higher expression in T-lep lesions, lower expression in L-lep lesions	(29)
DDs (FXIIIa)	Dermal	Higher expression in indeterminate leprosy lesions, lower expression in normal skin	(30)
DDs (FXIIIa)	Dermis	Positive expressions in different forms of leprosy, but no significant difference between polar forms of leprosy	(31)
CD1+CD83+ DC (CD1a, CD1b, CD1c)	Dermal granulomas	Higher expression in T-lep and reversal reaction leprosy lesions, lower expression in L-lep lesions	(33)
CD207+ DCs (CD207)	Epidermis and dermis	Higher expression in T-lep lesions, lower expression in L-lep lesions	(34)

*DCs, dendritic cells; LCs, Langerhans cells; pDCs, plasmacytoid dendritic cells; DDs, dermal dendrocytes; T-lep, tuberculoid leprosy; L-lep, lepromatous leprosy*.

Langerhans cells (LCs) are resident DCs located in the epidermis, which express the lipid-presenting molecules, CD1a and CD207 (Langerin). A number of studies have shown similar results, indicating that the number of LCs in the epidermis of T-lep lesions was significantly larger than in L-lep lesions (28, 29, 31). Since LCs gathered antigens and drove T cell responses through antigen presentation in the draining lymph node (35), these findings are consistent with the fact that cell-mediated immune responses dominate in T-lep, whereas L-lep is characterized by a humoral immune response. For indeterminate leprosy, no significant difference in LC expression was observed by the authors comparing indeterminate leprosy lesions and normal skin (30). But an increase in the numbers of LCs was observed in both T1R and T2R compared to non-reactional leprosy, which was consistent with the acute inflammatory reactions in leprosy reactions (36, 37).

Leprosy lesions and granulomas appear mainly in the dermis of patients, so DCs in the dermis of leprosy lesions have also received attention. Plasmacytoid DCs (pDCs), which express CD123 and dermal dendrocytes (DDs) characterized by the FXIIIa marker were studied in polar forms of leprosy. Similar to LCs, the presence of a relatively large number of pDCs and DDs was observed in T-lep lesions, but much fewer of these DCs were found in L-lep lesions (29–34). The expression of CD123 marker was also evaluated in T1R and corresponding BL and LL lesions. Using immunohistochemistry, real-time polymerase chain reaction (RT-PCR), and flow cytometry methods, the authors clearly showed that CD123 was significantly more abundant in T1R compared to BL and LL lesions (32).

CD207+ DCs were evaluated in both the epidermis and dermis in polar forms of leprosy by Hirai et al. (34). Compared to L-lep, the authors found a larger number of CD207+ DCs not only in the epidermis but also in the inflammation area of the dermis of T-lep lesions (34). However, the authors were not sure whether these Langerin-positive DCs in the dermis of leprosy lesions were LCs migrating from the epidermis or were another type of dermis-resident DCs (34). DC-specific intercellular adhesion molecule (ICAM)-grabbing non-integrin (DC-SIGN) is a C-type lectin expressed by subsets of DCs and macrophages and is an entry receptor for pathogens (38). Different from LCs, pDCs, and DDs, more DC-SIGN+ cells were found in L-lep other than T-lep (39, 40). Moreover, CD11c+ cells in PBMC of L-lep patients also showed higher expression of DC-SIGN (41), and peripheral monocyte of L-lep patients differentiated into DC-SIGN+ macrophages but not CD1b+ DCs with efficient antigen presentation function after TLR2/1 activation (39).

In contrast to the characterization of different subsets of DCs in leprosy lesions, fewer studies have focused on the specific roles of DCs in leprosy pathogenesis. CD1+ DCs induced by *M. leprae* antigens have been demonstrated to be efficient antigen-presenting cells for T cells (33, 42). LCs isolated from the epidermis of healthy volunteers also showed better efficiency than monocyte-derived DCs in presenting non-peptide antigens of *M. leprae* to T cells (43). Conversely, the recognition of *M. leprae* by DC-SIGN+ DCs showed immunosuppressive function by inducing IL-10 (44). However, the ability to present *M. leprae* antigens and the effect of *M. leprae* on DC differentiation were not investigated in pDCs or DDs. Moreover, the roles of DCs in leprosy pathogenesis are not restricted to antigen presentation, as DCs were also suggested to contribute to granuloma formation (45). Gene *MMP12*, part of the tissue remodeling network, was found to be connected to DCs in T-lep lesions using a cell type deconvolution-based gene expression analysis of leprosy lesions, which suggested the involvement of DCs in leprosy granuloma formation and/or maintenance (45).

#### Keratinocytes

Although the lesions of leprosy and the causal pathogens appear mainly in the dermis, the presence of *M. leprae* in the pilosebaceous unit and epidermis in BL and LL patients were repeatedly reported (46–48). Keratinocytes in the epidermis also show several immune responses in leprosy lesions or against *M. leprae* infection. Immunohistological studies have found that keratinocytes are strongly positive for HLA-DR antigens overlying T-lep lesions, but anti HLA-DR reactivity was negative for keratinocytes in L-lep lesions (49, 50). Further investigation on the interplay between keratinocytes and T cells indicated that HLA-DR+ keratinoc ytes could present *M. leprae* antigens to CD4+ Th1-like cells in a human leukocyte antigen (HLA) class II-restricted manner (51), consistent with the observation that HLA-DR-positive keratinocytes were abundantly overlying T-lep lesions. The intracellular adhesion between keratinocytes and lymphocytes, which is important for efficient antigen presenting, was also discovered in leprosy (52). In lesions of T-lep, as well as in reversal reactions and Mitsuda reactions, keratinocytes were found to express pronounced levels of ICAM-1, and lymphocytes contained within these lesions were positive for the ICAM-1 ligand lymphocyte function-associated antigen-1 (LFA) (52).

In addition to acting as effective antigen-presenting cells, keratinocytes were also found to defend against *M. leprae* directly using antimicrobial means. Compared to normal skin, strong expression of nitrotyrosine and iNOS were observed in keratinocytes in granulomas from borderline leprosy patients (53). Higher expression of human beta-defensin 3 was found in lesions from patients displaying the type 1 reaction compared to leprosy patients negative for the type 1 reaction (54). Further studies indicated that keratinocytes, rather than macrophages, upregulated human beta-defensin 2 and human beta-defensin 3 in response to *M. leprae* stimulation (54). The *in vitro* phagocytosis of *M. leprae* by keratinocytes was shown for the first time by Lyrio et al. (55), who also demonstrated that keratinocytes infected by *M. leprae* increased the expression of cathelicidin and tumor necrosis factor (TNF)-α.

## Adaptive Immune Responses in Leprosy

### The Th1/Th2 Paradigm

Although the role of innate immune cells in leprosy pathogenesis cannot be neglected, it appears that the responses of T cells determine the outcome in leprosy development. In a murine model, Th1 cells that produced IL-2 and IFN-γ could prime macrophages to the microbicidal M1 polar state and produce a restricted form of the disease. In contrast, Th2 cells that produced IL-4 and IL-5 inhibited the microbicidal function of macrophages, resulting in the progressive form of the disease (56). In tuberculoid patient lesions, it was found that cytokines IL-2 and IFN-γ showed remarkably higher expression while IL-4, IL-5, and IL-10 were more abundant in lepromatous lesions (56). This cytokine pattern in leprosy lesions is very similar to the murine Th1/Th2 model. In addition, cytokine profiles of PBMCs from leprosy patients also showed a polar pattern according to leprosy type (57, 58). Although half of PBMCs from subjects showed non-discriminating Th0 responses upon *M. leprae* antigen stimulation, the remaining L-lep presented Th2 responses while T-lep showed Th1 cytokine responses (57). But the Th1 or Th2 responses is not irreversible in patients, for example, the shifting from a Th2 profile to a Th1 profile with the increased production of IFN-γ and CXCL10 is a prominent character of T1R (59–61).

The Th1/Th2 paradigm can explain the manifestations and histopathology of the two polar forms of leprosy. In localized TT, the cell-mediated immune response is strong and the bacilli are rarely observed, whereas in disseminated LL, the humoral response dominates and the bacilli load is high. In addition, during the past few decades, several other smaller lymphocyte subsets have been described in leprosy lesions, which may be involved in leprosy pathogenesis, especially in the shaping of host immune responses to infection.

### The Reciprocal Relationship Between Regulatory T Cells and Th17 Subsets

Regulatory T cells (Tregs) and IL-17-producing Th17 cells are functionally and developmentally reciprocal to each other (62–64). Naive T cells develop into Tregs in the presence of transforming growth factor (TGF)-β, while the combination of TGF-β and IL-6/IL-21 introduce naive T cells into Th17 (62–64). Tregs with tolerance/immunosuppression functions are the primary mediators maintaining peripheral tolerance and are essential for the prevention of autoimmune diseases and chronic inflammatory diseases (65). However, Tregs may also suppress the appropriate host immune responses against infections (65). IL-17-producing Th17 cells with immunity/inflammation functions are a recently discovered and characterized subset of effector T helper cells, which have a reciprocal relationship with Tregs in subsets of developmental programs (63).

IL-10 producing CD4+CD25+FoxP3+ Tregs were analyzed in LL/BL, BT/TT, and healthy controls by Kumar et al. (66) who found that the expression of Tregs was higher in patients than in healthy controls and that LL/BL patients showed the highest expression of Tregs, which was consistent with the anergy of T cell responses in L-lep (66). Similarly, a high frequency of TGF-β secreting CD4^+^CD25^+^ FOXP3^+^ Tregs was also observed in L-lep (67). IL-35 is another suppressive cytokine and was shown to increase significantly in CD4^+^CD25^+^ Tregs of leprosy patients as compared to healthy controls (68). In contrast, Th17-related cytokines, chemokines, transcription factors, and Th17 cells showed higher expression in T-lep compared to L-lep (69, 70). The reciprocal relationship between Treg and Th17 cells has also been observed in leprosy (71). After *M. leprae* antigen stimulation, a higher frequency of Tregs was found in PBMCs of BL/LL patients, while a conversely higher frequency of Th17 cells was found in PBMCs from BT/TT patients (71). But the reciprocal relationship between Treg and Th17 was not irreversible, a recent study has shown that Tregs from leprosy patients could be converted to IL-17 producing Th17-like cells by rIL-23, suggesting a new way to overcome the immunosuppression in leprosy patients, especially in L-lep (72).

However, the reciprocal relationship between Treg and Th17 may be not applicable in leprosy reactions, especially for T1R. Since T1R and T2R are both acute inflammation reactions, it is reasonable to discover a higher frequency of Th17 in both T1R and T2R compared to non-reactional leprosy considering the inflammation functions of Th17 cells (73–75). Comparing to other forms of leprosy and healthy controls, T2R showed lowest circulating Treg frequency, which was consistent with the high Th17 frequency in T2R (58, 76). But it is not the case for T1R, several studies have demonstrated that T1R showed a significantly higher frequency of Treg compared to non-reactional leprosy in both PBMC and skin lesions (58, 74, 76–78). One possible explanation for the increase of Treg in T1R is that the increase of Treg in T1R is a self-protection mechanism to reduce the tissue damage caused by the exacerbated cell-mediated immune responses.

### Th9 and Th22

In the presence of IL-4 and TGF-β, Th0 lymphocytes differentiate into Th9 lymphocytes, which preferentially produce IL-9, IL-10, and IL-21 (79). The Th22 lineage is characterized by the production of IL-22 and several fibroblast growth factors and also expresses the skin homing receptors CCR4 and CCR10, suggestive of the possible roles of Th22 in skin diseases (80). These two subsets of T helper cells were both studied by quantitative determination of their signature cytokines. IL-9 was found to be expressed more highly in TT lesions compared to LL lesions (81), consistent with the early finding that IL-9 could promote anti-*M. leprae* cytotoxicity (82). In contrast, the expression of Th22 signatures IL-22 and fibroblast growth factor basic (FGF-b) was higher in LL lesions compared to TT lesions (83).

### γδ T Cells

**γδ** T cells is one of three lymphocyte lineages and only constitutes a very small proportion of lymphocytes as compared to the conventional αβ T cells and B cells (84). But γδ T cells have various immune functions and have been suggested to play roles in infectious diseases including leprosy (84, 85). In the case of leprosy, at least two different roles of γδ T cells have been suggested, namely, contributing to leprosy reactions and immunosuppression. Modlin et al. (86) firstly observed a significant increase of γδ T cell frequency in granulomatous reactions of leprosy. Following studies also confirmed that γδ T cell frequency increased significantly in Mitsuda reaction, a form of delayed hypersensitivity in leprosy (87, 88). But underlying mechanism of the contributions of γδ T cells to leprosy reactions was unknown until recently. Saini et al. (89) found that γδ T cells showed a higher frequency in both T1R and T2R reaction patients as compared to stable patients, and they also showed that these γδ T cells produced a notable amount of IL-17 and IFN-γ, which may explain the mechanism that γδ T cells contribute to leprosy reactions (89). In 2004, Sridevi et al. (90) reported the high level of γδ T cells in L-lep patients. And then the immunosuppressive role of γδ T cells was systematically studied (91). CD4+TCRγδ+FoxP3+ cells were observed to be significantly increased when moved from healthy controls and T-lep to L-lep patients, and the immunosuppressive nature of CD4+TCRγδ+FoxP3+ cells was also evidenced by *in vitro* experiments (91).

### B Regulatory Cell

Humoral immunity in leprosy was suggested to be ineffective in pathogen elimination because *M. leprae* could survive and multiply in humoral immune-dominated L-lep despite the greater antibody responses in L-lep (92, 93). Therefore, B cell is the least cell population to be considered in leprosy pathogenesis studies, although it is a major immune cell population with antibodies secretory and antigen presentation functions. But recent studies on B cells showed that three subsets of B regulatory cells (Bregs) which showed immunosuppressive functions may play important roles in leprosy pathogenesis (68, 94–96). IL-35 producing Bregs showed a higher frequency in leprosy patients compared to healthy controls, and a positive correlation between IL-35 and bacteriological index was also observed (68). The second reported subset is IL-10 producing Breg, and this subset was also demonstrated to show an increased frequency in PBMCs of leprosy patients as compared to healthy controls (96). Furthermore, the authors showed that IL-10 producing Breg could convert effector T cells to Tregs and enhance the function of Treg (96). Besides IL-10 and IL-35 producing Breg, tissue-like memory B cells with immunosuppressive functions were also reported to be more abundant in L-lep comparing to T2R patients (95). These studies on Breg clearly showed that immunosuppressive functions of Breg may play an important role in the immunopathogenesis of leprosy.

## The Hereditability of Leprosy Genetic Risk Factors

### Association Between Leprosy and Innate Immune-Related Genes

Skin is the first physical barrier of human hosts against microbial invasion. Filaggrin, encoded by gene *FLG*, is the main constituent of keratohyalin granules and is indispensable for the proper function of the epidermal barrier. And loss-of-function mutations of *FLG* have been demonstrated to cause skin barrier deficiency and increase the risk to bacterial infection (97, 98). In an exome-wide association study, rs146466242 (K4022X), a loss-of-function mutation of *FLG*, was found to be associated with leprosy in Chinese populations (99). This finding raised the speculation that the impaired skin barrier might be an important route of *M. leprae* and other bacterial infections. But further genetically or biological experiments were needed to confirm this speculation. Genetically, more studies on the association between other infectious skin diseases and loss-of-function mutations of *FLG* could be performed. Biologically, the invasion of *M. leprae* and other bacteria into skin could be performed using mouse carrying *flg* loss-of-function mutations and human epidermal model constructed from keratinocyte with *FLG* loss-of-function mutations.

Innate immune cells recognize invading pathogens through receptors for pathogen-associated molecular patterns and then initiate specific innate immune responses. Both extracellular and intracellular pattern recognition receptor-coding genes have been found to be associated with leprosy. In a CGAS, functionally relevant coding single-nucleotide polymorphisms (SNPs) of *TLR1*/*TLR2* were studied in 543 Bangladeshi leprosy patients and 842 healthy controls, and the polymorphism N248S was found to be associated with leprosy (100). Also, in the first large-scale GWAS of leprosy, it was discovered that the intracellular pattern recognition receptor gene *NOD2* was associated with leprosy in Chinese (101). These findings were replicated in a case-control CGAS study using 933 patients in Nepal (102). The role of *NOD2* in leprosy immune response was also functionally confirmed by cell-based experiments. In monocyte, recognition of NOD2 by leprosy muramyl dipeptide could induce the expression of IL-32, which regulates the differentiation from monocyte to CD1b+ DC (42, 103). But knowledge about effect of variants on function of *NOD2* was still absent in leprosy.

Autophagy and phagocytosis are important defense mechanisms of the host innate immune system against intracellular pathogens (104, 105). Intron variant rs2275606 in gene *RAB32*, encoding a critical molecule required for the biogenesis of lysosomal-related organelles (106), was identified as associated with leprosy in a GWAS using Chinese Han population subjects (107). In a *Listeria monocytogenes*-infected DC model, Li et al. (108) provided direct evidence that Rab32 was part of a complex which encompassed bacteria and controlled the intracellular growth of *L. monocytogenes*. Additionally, the lysosome and endosome membrane protein-encoding gene *SLC29A3* was also discovered by this research group to be a leprosy susceptibility gene (99). These findings indicated the involvement of autophagy or phagocytosis in leprosy pathogenesis. Moreover, two other genes that regulate autophagy, *LRRK2* and *IRGM*, were found to be associated with leprosy (109, 110). In a zebrafish model, the *LRRK2* mutant showed a weakened immune response to *Mycobacterium marinum* infection, functionally confirming the role of *LRRK2* in infectious diseases (111).

To directly kill invading pathogens is one important part of innate cell functions. Several genes related to microbicidal functions have been identified as leprosy susceptibility genes. The exonic variant rs13259978 in *SLC7A2* was found to be associated with leprosy (112). Since *SLC7A2* is an important component of the classic nitric oxide antimicrobial pathway in macrophages, this finding suggested the involvement of the nitric oxide microbicidal pathway in leprosy. Another classic antimicrobial pathway, the vitamin D antimicrobial pathway has also been suggested to be involved in leprosy. Early in 1999, the TaqI polymorphism in the 3′ region of the vitamin D receptor (VDR) gene *VDR* was reported to be associated with leprosy in Indians (113). Furthermore, a silent change in codon 352 of the *VDR* gene also showed association with leprosy in Malawians (114). However, consistent results were not obtained in a case-control study including 933 leprosy patients and 101 controls in Nepal, in which the TaqI polymorphism of *VDR* showed no association with leprosy (115). As explained by the authors, this negative finding might be caused by population heterogeneity, different sample size, or alteration in the virulence of *M. leprae* in different geographical regions (115). Association between *OPA1* common variants and L-lep was observed in Han Chinese of southwest China (116). Gene *OPA1* encodes an inner membrane protein of mitochondria, which suggests a third antimicrobial pathway in leprosy, the mitochondrial antimicrobial pathway that functions by generating reactive oxygen species (116). In addition to these three antimicrobial pathways, several genes encoding products that have microbicidal functions were also found to be associated with leprosy, such as the lysosomal cysteine protease encoding gene *CTSB* (cathepsin B) (117), the antimicrobial peptide encoding gene *DEFB1* (beta-defensin 1) (118) and the *IFNG* gene (119, 120).

HLAs play key roles in the presentation of antigens to T cells and are indispensable for adaptive immune responses. Several members belonging to the classical class I and class II HLA genes have been shown to be associated with leprosy. Leprosy-associated class I HLA genes include HLA-A^*^28 in Mestizo populations (121) and HLA-B^*^15 and HLA-C^*^05 in the Brazilian population (122). Leprosy-associated class II HLA genes include *HLA-DQA1* in Brazilians (123), HLA-DQB1^*^06 and HLA-DQB1^*^07 in Mestizo populations (121), HLA-DRB1^*^11 in Brazilians (124), HLA-DRB1^*^1501 in Chinese and Indians (125, 126), and HLA-DRB1^*^1501 in Indians (125, 126). In addition, other antigen-processing and presentation-related genes were found to be associated with leprosy, such as the *MICA* gene (127), *MICB* gene (127), *HLA-G* gene (128), and *TAP1* gene (129). Interestingly, even in the same population, some HLA genes showed susceptibility to leprosy and others showed resistance to leprosy, which indicated that some HLA molecules could activate T cells by presenting *M. leprae* antigens, while others are responsible for the T cell anergy in leprosy (121).

The complement system is an important component of the host innate immune system, and several gene members of the complement system were identified as associated with leprosy. In 2007, the association between *MBL2* and leprosy was revealed by a study examining polymorphisms at the promoter and exon 1 regions of this gene (130). This group also found that *FCN2* was associated with Brazilian leprosy patients (131). The mannose binding lectin encoded by *MBL2* and ficolin-2 encoded by *FCN2* both have essential roles in the lectin complement pathway. These two findings were subsequently confirmed by a candidate loci association study using Han Chinese, in which the authors found that genetic variants of *FCN2* and *MBL2* genes conferred susceptibility to leprosy (132). Moreover, this study also found two variants of the *CFH* gene, which showed significant associations with leprosy, indicating the involvement of the alternative complement pathway in leprosy pathogenesis (132).

### Leprosy-Associated Adaptive Immune-Related Genes

Compared to innate immune-related genes, far fewer adaptive immune-related genes have been found to be associated with leprosy. Nevertheless, various genes involved in Th1, Th2, and Th17 differentiation and responses were found to be associated with leprosy. In a multiple-stage candidate susceptibility loci association study, *IL18R1* was identified as a leprosy risk gene in a Chinese population. *IL18R1* encodes the receptor of IL-18, which can promote Th1 responses to *M. leprae* (133). Also in a Chinese population, the 590T/C polymorphism of *IL4* was found to be associated with leprosy, which indicated Th2 involvement in leprosy since IL-4 is a typical Th2 cytokine. Two IL-23/Th-17 pathway genes, *IL12B* encoding the heterodimeric subunit of IL-23 and *IL23R* encoding the IL23 receptor, were identified as leprosy susceptibility genes in different populations (107, 133–136). The Janus kinase (JAK)–signal transducer and activator of transcription (STAT) signaling pathway is the downstream pathway of IL-23 signaling, and two key genes of this pathway, *TYK2* and *SOCS1*, were also found to be associated with leprosy (99, 137). These findings strongly suggest the involvement of Th17 responses in leprosy. Moreover, *TNFSF15*, which encodes a mediator of the switch from Th1 to Th2 phenotype, was also found to be a leprosy susceptibility gene (101, 138).

### Roles of Cytokine Gene Polymorphisms in Leprosy

**Figure 1:**
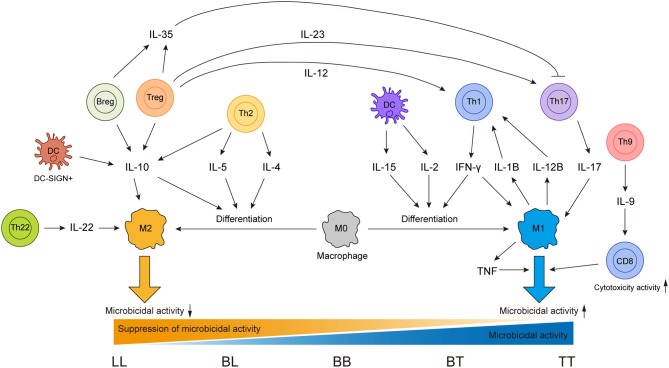
Sources of different cytokines and effects of these cytokines on different immune cells in leprosy immune responses.

Cytokines are secreted by various immune cells and play essential roles in shaping the immune responses in leprosy and even can drive the conversion between functionally antagonistic cells (Figure 1). Therefore, association between cytokine gene polymorphisms and leprosy has been one of the focuses in leprosy genetic study. In addition to *IL4* and *IL12B* mentioned above, other cytokine genes that may play important roles in leprosy immune responses were also genetically investigated. The tumor necrosis factor (TNF)–lymphotoxin-α (LTA) locus was found to be leprosy-associated by both linkage analysis and SNP scanning of this region (139, 140). This region encodes pleiotropic cytokines TNF and LTA, which have various immune regulatory functions. Moreover, the most frequently involved SNP of *TNF* in infectious diseases, *TNF*-308 G>A (rs1800629), has been widely studied in leprosy with different ethnicities, including Nepalese, Brazilians, and Indians (115, 141, 142). However, these studies obtained inconsistent results for the association of *TNF*-308 G>A with leprosy, which inspired a meta-analysis of 14 studies on this topic (143). The meta-analysis found that no association was observed in the overall population or in Asians, but *TNF*-308 G>A showed a protective effect against leprosy risk in the Latin American population (143). For *LTA* of this region, a fine linkage disequilibrium mapping study discovered that *LTA*+80 A allele was significantly associated with leprosy risk (144). TNF and LTA were also suggested to play essential but different roles in the regulation of leprosy granuloma formation (145).

IFN-γ can shape the immune responses in leprosy by activating innate immune cells (15); studies focused on the SNP +874 A/T (rs2430561) have been conducted in leprosy. In the Brazilian population, a protective effect of *IFNG* +874 A/T was observed which was confirmed by the higher level of IFN-γ produced by PBMCs (119). A meta-analysis using data from different populations also found that *IFNG* +874 A/T showed a protective effect for leprosy (146). IL-10, a cytokine with immunosuppressive properties secreted by monocyte and lymphocyte lineages, has been demonstrated to play roles in the development of L-lep (15, 96). Variant−819 C/T (rs1800871) in the promoter region of gene *IL10* was repeatedly found to be associated with leprosy (142, 147). And two meta analyses focusing on *IL10* variants both confirmed the significant susceptibility association between−819 C/T (rs1800871) and leprosy (148, 149). These results suggested that these variants of *IFNG* and *IL10* might regulate the immune response and even the disease progression of leprosy.

### Genes Associated With Leprosy Reactions

Although immunological studies have made significant progress in the pathogenesis of leprosy reactions, the intrinsic mechanism involved in the onset of leprosy reactions remains largely unknown. And host genetic factors have also been suggested to contribute to the developing of leprosy reactions (7). A comprehensive review on the genetics of leprosy reaction has well summarized the studies on the association between leprosy reactions and genes *TLR1, TLR2, NOD2, IL6, C4B, VDR*, and *SLC11A1* (7). Therefore, here we reviewed several important advances of leprosy reactions genetic studies made in recent years.

TNFSF15 and TNFSF8 are both members of TNF-like molecules. TNFSF15 play roles in the switch from Th1 to Th2 cells (150, 151), while TNFSF8 impact the balance between Th1 and Th2 cells (152). Since immune responses in T1R is characterized by the switch from Th2 to Th1, it is reasonable to study the association between T1R and these two genes. To evaluate the association of these two genes with T1R, the fine mapping of the *TNFSF15-TNFSF8* locus was performed separately in Vietnamese and Brazilians populations. And the results indicated that *TNFSF8* was genetically associated with T1R in these two geographically and ethnically distinct populations, but not *TNFSF15* (153). This study suggested that TNFSF8 might be a mediator of excessive inflammatory responses, which needs to be further confirmed. But the strong associations of *TNFSF15/TNFSF8* variants rs6478108 and rs7863183 with T1R in Vietnamese patients were not observed in the Brazilian patients (153). Considering the significant difference in the age of diagnosis between Vietnamese and Brazilian populations, this research group further validated three regulators of the *TNFSF8* gene transcription rs3181348, rs6478108, and rs7863183 in additional two Vietnamese and two Brazilian populations (154). They found that the associations with T1R for rs7863183 and rs3181348 were observed in all diagnosis age groups, but for rs6478108 was diagnosis age dependent which suggested that the regulation of *TNFSF8* transcription might be age dependent (154).

The involvement of HLA complex in leprosy has been demonstrated immunologically and genetically, but genetic studies of HLA genes in borderline leprosy and leprosy reactions were rarely reported. de Souza-Santana et al. (122) genotyped the HLA class I (A^*^, B^*^, and C^*^) and class II (DRB1^*^ and DQB1^*^) loci in a cohort consisting of 202 borderline leprosy (BT, BB, BL) patients of which 94 had T1R and 478 healthy controls (122). Their results demonstrated that HLA-C^*^05, HLA-DRB1^*^07, and HLA-DQB1^*^02 were genetically associated with borderline leprosy, while HLA-B^*^15 showed a significantly high frequency in T1R (122). Since the association between HLA and T1R is rarely reported in the literature, these results need further confirmation in different populations.

T1R is the main cause of nerve damage in leprosy patients (155). Causal genes of Parkinson's *LRRK2* and *PRKN* were found to be associated with T1R in a genewise enrichment analysis including 63 coding variants of seven genes (156). Moreover, gain-of-function mutation LRRK2 R1628P was functionally shown to be protective for T1R by experiments performed in gene-edited RAW cells, and similarly, rare non-synonymous variants of *PRKN* were also functionally determined to be T1R risk factors (156). This study not only functionally linked the genetic associated mutations with nerve damage in T1R but also provided a good example to follow in the future for studying the pathogenesis of leprosy and T1R-associated variants.

### Other Leprosy-Associated Genes

Genetic studies have also discovered other leprosy-associated genes, such as genes related to lipid metabolism, nerve damage, oxidative stress, and ubiquitin-mediated proteolysis. The accumulation of lipids in macrophages is thought to be beneficial for mycobacterium survival, and variants of two lipid metabolism related-genes, *ALDH2* and *APOE*, were shown to confer risk for leprosy (112, 157). Although nerve damage is frequently observed in leprosy patients, the association between leprosy and nerve-related genes has rarely been reported. *SYN2* encodes a neuronal phosphoprotein, which is a member of the synapsin gene family. In a large-scale genome-wide association and meta-analysis study, rs6807915 near *SYN2* was identified as a susceptibility locus for leprosy. This finding suggested a role for synapsin-2 in the progression of infection from mycobacteria to the nerves (117). Unexpectedly, two oxidative stress-related genes, *SOD2* and *HIF1A*, were also found to be associated with leprosy, which may be explained by the possibility that oxidative stress is a means of defense used by *M. leprae*-infected cells (158, 159). Early in 2004, a genome-wide linkage scan study reported the association between the 5′ regulatory region of *PARK2*/*PACRG* and Vietnamese leprosy, which was confirmed in Brazilian leprosy patients (160). Combining this genetic finding with the expression of *PARK2* and *PACRG* in macrophages and Schwann cells, the authors pointed out that ubiquitin-mediated proteolysis might function in leprosy pathogenesis, but this association signal was not observed in Indians or Chinese (161, 162).

### Expression of Human Genes in Leprosy

Plenty of literature have reported the genetic association between human genes and leprosy at DNA level using variants genotyping, while only a few studies have focused on the association between expression of human genes and leprosy. Studies on the expression of human genes in leprosy have been performed using various specimen types from different clinical forms of leprosy patients. A comprehensive genome-wide analysis of human mRNA expression in skin lesions of all forms of leprosy (TT, BT, BB, BL, LL, T1R, and T2R) was performed using microarrays, RT-PCR, and immunohistochemistry, and different forms of leprosy showed some unique differentially expressed genes, such as *GPNMB, IL1B, MICAL2*, and *FOXQ1* in T1R and *AKR1B10, FAM180B, FOXQ1, NNMT, NR1D1, PTX3*, and *TNFRSF25* in T2R (26). Although this finding revealed the complexity of this disease from a molecular point of view with solid evidences, few of discovered genes could be used to explain specific pathogenesis due to that these genes could hardly be linked to a specific cell type of leprosy lesions (26). In a cell-type deconvolution analysis of leprosy lesions transcriptome data, Inkeles et al. (45) found roles of some cell-defined genes in leprosy immunopathology. For example, the authors demonstrated granuloma formation/inflammation roles of *MMP12* in DCs of T-Lep/T1R and chemotaxis roles of gene *CXCL1, CXCL5*, and *CCR2* associated with neutrophil in T2R (45). Besides transcriptome profiling of leprosy lesions, transcriptome data of whole blood cells stimulated with *M. leprae* were also an efficient way to reveal gene expression signature related to leprosy. An elaborated analysis of transcriptome response of whole blood from former T1R and T1R-free patients to *M. leprae* sonicate identified a T1R gene set signature mainly consisting of innate pro-inflammation genes, such as *CCL2, IL1A*, and *IL1B* (163). This study suggested that defect in the regulation of innate pro-inflammation genes contributed to risk of T1R onset (163). A major problem of studies on expression of human genes in leprosy is that in rare cases, genetic associated genes were observed to be up or downregulated in any form of leprosy as compared to corresponding controls. Considering the high complexity in cell types of leprosy lesions, this problem may be caused by that genes *per se* are heterogeneously expressed in different types of cells.

## Discussion

**Figure 2:**
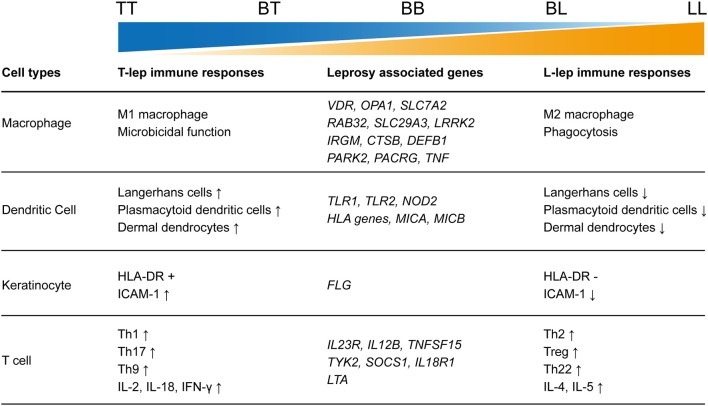
Summary of leprosy-associated genes and immune responses to leprosy.

As an infectious disease, the landscape of immune responses in leprosy has been widely studied using skin lesions or PBMCs from leprosy patients. Various innate and adaptive immune cells have been shown to be involved in the immune responses to leprosy (Figure 2). Particularly, immunological studies have revealed remarkable differences between the two polar forms of leprosy (Figure 1). However, due to the lack of an efficient leprosy animal model, immunological studies were unable to delve deep into the gene or molecule level and could not explain why leprosy occurs only in certain populations and how leprosy develops into spectrum-like clinical manifestations. Genetic studies give us another opportunity to uncover the molecular mechanism underlying immune responses. Since most leprosy-associated genes are immune related (Figure 1), it is reasonable to speculate that changes in gene functions caused by leprosy-associated variants may make the immune system of the host unresponsive, disordered, or over-reactive when exposed to *M. leprae* infection, which contributes to the onset and spectrum development of leprosy. However, many leprosy-associated genes or variants have not been confirmed by biological experiments, leaving these genetic findings unsubstantiated.

The combination of immunological and genetic approaches is a promising way to explain leprosy pathogenesis more clearly and deeply. That is to say, we can fine-map the leprosy-associated gene loci and find the candidate causal variants and then explore the effect of these candidate causal variants on protein function and immune responses. The fine-mapping can be performed using large cohorts by next-generation sequencing, and cells from volunteers who carry candidate variants or gene-edited cells may be subjects for studies on the effects of these candidate variants on protein functions and immune responses; however, analysis may still be difficult because of the lack of an *in vivo* or *in vitro* leprosy model. The co-culture of innate and adaptive immune cells may be an option for an *in vitro* leprosy model, but up to now no such reported *in vitro* model has been widely accepted and applied. A mouse model for leprosy also failed due to the immune differences between mice and humans. A humanized mouse model may be a solution to this problem. Studies on the effects of disease-associated variants on protein function and immune response is not only important for the elucidation of leprosy pathogenesis but may also provide clues for genetic diagnosis and more precise therapeutic interventions.

## Author Contributions

FZ and HL designed this work and revised the manuscript. ZM wrote the manuscript.

### Conflict of Interest

The authors declare that the research was conducted in the absence of any commercial or financial relationships that could be construed as a potential conflict of interest.
